# Factors influencing the utilization of focused antenatal care services in Malindi and Magarini sub-counties of Kilifi county, Kenya

**DOI:** 10.11604/pamj.supp.2016.25.2.10520

**Published:** 2016-11-26

**Authors:** Dorah Chorongo, Fredrick Majiwa Okinda, Eric Jimmy Kariuki, Emily Mulewa, Fredrick Ibinda, Samuel Muhula, George Kimathi, Richard Muga

**Affiliations:** 1 Ministry of Health, Kenya; 2 Amref Health Africa in Kenya; 3 Unicef; 4 KEMRI-Wellcome Trust Research Programme, Kenya; 5 Uzima University, Kenya

**Keywords:** Attendance, antenatal care, health service delivery

## Abstract

**Introduction:**

Globally, pregnancy related complications contribute to more than half of the deaths among women annually. Antenatal care (ANC) is important for the prevention of maternal and fetal mortality and morbidity. This study identifies the socio-demographic and economic characteristics; knowledge and attitude and; health service provision for focused antenatal care (FANC) services.

**Methods:**

A cross-sectional comparative study conducted among 385 women of reproductive age who visited 5 public health facilities in Malindi and Magarini Sub Counties. Data collection was conducted between June 2013 and September 2013 through structured questionnaires, Key Informant Interviews (KIIs) and Focused Group Discussions (FGDs) and analyzed through descriptive summary statistics and test of associations of the various variables using chi square.

**Results:**

About 35% of women sought 1st and 2nd ANC services at the health facilities. These women went ahead to complete the recommended 4 minimum visits as recommended by World Health Organization (WHO). Compared to Catholics, Muslims were less likely to attend a rural health facility (Odds ratio (OR) = 0.25, 95% Confidence Interval (CI) 0.10, 0.62, p=0.003). According to education levels, those with secondary (OR=0.07, 95% CI 0.03, 0.18, p<0.001) or tertiary (OR=0.09, 95% CI 0.03-0.17, p<0.001) levels of education were less likely to seek FANC at rural facility. Women seeking rural ANC services started at 2nd or 3rd trimester (OR=5.40 95% CI 2.97-10.06, p<0.001) while those in urban setup start at 1st trimester. Among the women who were aware of FANC, only 27% utilized its services. Long waiting hours, unavailability of services, and departmental disharmony were major causes of dissatisfaction to mothers visiting the facilities.

**Conclusion:**

Utilization and awareness of FANC services in both rural and urban health facilities among women in Malindi and Magarini Sub Counties continues to be low which is associated by socio-demographic characteristics, and health facility inefficiencies. Thus there is need to standardize services across health facilities and increase awareness on FANC in both rural and urban.

## Introduction

Worldwide pregnancy related complications contribute to more than half of deaths among women annually. According to WHO, about 90-95% of these come from developing countries [1]. The launch of the Safe Motherhood Initiative (SMI) was seen as a major milestone in the race to reduce the burden of maternal mortality throughout the world, particularly in developing countries. It issued a call to action to reduce maternal mortality and morbidity by one half by the year 2000 [2].

Focused Antenatal Care (FANC) is a new model of antenatal clinic attendance introduced by WHO that is goal-oriented, which reduces the number of required antenatal visits to four, and provides focused services shown to improve maternal outcomes. While being a strategy it is also an important determinant of safe delivery which provides an opportunity for women to be educated to recognize and act on symptoms associated with potentially serious conditions like pre-eclampsia or a malaria infection and obstructed labour as a strategy for reducing maternal mortality. The World Health Organization recommends that a woman without complications should have at least four visits to get sufficient prenatal care [1]. Prenatal care is more likely to be effective if women begin to receive care in the first trimester of pregnancy and continue to receive care throughout pregnancy [3]. However, much work has yet to be done to assure maternal health for women worldwide. In order to reduce life-threatening risks and reduce mortality, good-quality maternal health services by trained health workers must be available and must be used. Therefore, safe motherhood strategies must be comprehensive in nature; even when quality health services are available, other limiting factors can get in the way of women using these services, such as social, economic and cultural factors [1].

WHO recommended Focused Antenatal Care (FANC) package that promotes the implementation of interventions to address the most prevalent health issues affecting pregnant mothers and newborns [4]. The Kenya Ministry of Health (MOH) has designed new guidelines for FANC services, placing emphasis on 4 ANC visits that focus on antenatal care, birth planning and emergency preparedness. These visits are now used as an entry point for a range of other reproductive health services, thus promoting comprehensive integrated service delivery [5]. Proven effective antenatal interventions include serologic screening for syphilis, provision of malaria prevention, anti-tetanus immunization, and prevention of mother-to-child transmission of HIV among others.

Malindi and Magarini Sub-counties has implemented FANC activities as stipulated by the National guidelines. However, there is a marked success in the first and second visits standing at (90% and 80% respectively) while the third and fourth visits stand at 50% and 35.2% respectively [6]. Various reasons have been given that mothers are starting clinics late and end up not completing the four ANC visits despite the efforts being put in place on health education. This warrants the study of the underlying factors for late prenatal attendance and incomplete utilization of the services offered during FANC. The study therefore sought to establish socio-demographic factors associated with utilizing focused antenatal care services determine the level of awareness and perception of clients towards frequency and timing of FANC services, the proportion of pregnant women who utilize FANC services correctly as stipulated in the FANC strategy and the health service provision factors that influence frequency and timing in the uptake of FANC services.

## Methods

### Study design

A descriptive cross-sectional study was conducted in the form of face-to-face structured interviews in Malindi and Magarini Sub-Counties of Kilifi in Kenya between June 2013 and September 2013. A total of 10(34.4%) of GoK health facilities were randomly selected from 4 divisions in Malindi and Magarini Sub-Counties which included 6 dispensaries, 3 health centers and 1 sub-county hospital in each division were included in the study. In these health facilities, a total of 385 women attending ANC services and those with children <5 years were interviewed after consenting. Qualitative data collection was conducted through focused group discussions with women who attended ANC while key informants interviews done to health facility in-charges.

### Study setting and participants

The study was conducted in Malindi and Magarini Sub Counties of Kilifi County in Kenya in Coast Region in four Divisions of Malindi, Magarini, Marafa and Lango Baya. Malindi and Magarini Sub-Counties borders Taita-Taveta to the west, Tana Delta to the northwest and the Indian Ocean to the east. It lies between latitudes 2.2o and 4o south and between longitudes 39o and 41o east with a total population estimated to be 436,371. The Sub-County is cosmopolitan mainly composed of the Mijikend as followed by Swahili/Bajuni tribe. Immigrants from upcountry comprise about 10% of the total population and Europeans consist of a smaller number (5%) [7]. The area which is majorly rural (86%) has the main religions being Christianity and Islam (40%). The Sub-County is also characterized by a shortage of health care workers with a nurse to population ratio of 1:8594 and high infant mortality rate of 71/1000 [8]. The study involved mothers bringing their children (<5 years) to health facilities for immunization and weighing, women coming for family planning services and pregnant women attending ANC services.

### Sampling procedure

Systematic sampling was applied to select respondents among women who came for ANC, PNC

Family planning and those bringing their children (<5 years) for immunization. The sampling procedure involved choosing every third woman in the queue as a respondent. Purposive sampling was applied to choose the KII participants. A total sample of 385 respondents was arrived at 95% confidence level and 5% degree of accuracy (Mugenda Mugenda 1998).

### Data collection techniques

A semi structured interview schedule (questionnaire) was administered to all the women who met the inclusion criteria above. KII guide was used to get qualitative information from a sample of health workers and FGD guide was used to gather qualitative data. Six (6) KIIs were conducted to cover all the 4 divisions in the study area where 4 were distributed in dispensaries across the divisions, 2 at the Sub county referral hospital while the 1 FGD were done in the Sub-County hospital for urban set up and one for rural facility.

### Ethical considerations

Informed consent was sought from willing respondents before data collection. Letter of Authorization was also sought from Kenyatta University, Kenya since the study was not going to be invasive in nature.

## Results

### Socio-demographic and economic characteristics

A total of 385 respondents were interviewed. The average age was 25 (Standard error: 0.27) years. According to marital status, 317(86%) were married (from urban 70% and rural 90.8%), 33(9%) were single (17.2% in urban and 4.8% in rural) and 20(5%) of them were either widowed or divorced. About 245(63.7%) were self-employed, 52(13.6%) had formal employment while 88(22.7%) were unemployed. Most of the mothers (73.5%) were of Christian faith (where 61.9% were Protestants and 11.53% were Catholics) while 12.3% were Muslims and 14.2% either pagans or had no religion. Majority in the urban setup were Protestants (59.1%) followed by Muslim (28.0%) then Catholic (10.8%) and others 2.2% in that sequence. While in the rural setup, majority were Protestants (63.1%) followed by other religions (16.5%), Catholic (12.2%) and the Muslim (7.9%).

Slightly more than half of the women 228(59.5%) had primary level education, 51(13.3%) had post primary education, 80(20.9%) had no education and 24(6.3%) had received tertiary education and above. As for their spouses, majority 235(61%) were of primary education level. About 293 women (78.76%) said they had no family and household related chores that hindered them attend ANC, while 79 women (21.24%) agreed that family responsibilities and especially finances prohibited them from attending ANC visit.

### Level of awareness and perceptions of mothers

There was a high level of awareness at 91.5 % (348) reported having heard of FANC. The major independent predictor for lack of awareness was being unemployed (OR=9.94, 95%CI 2.02, 48.94, p=0.005). In the rural area, awareness was found to be higher among Catholics (77.8%) compared to Muslims (42.1%) while in the Urban areas 22% of Muslims and 57.9% of Catholics exhibiting awareness of FANC. The level of awareness was higher among the educated in both rural (71.7%) and urban (28.3%) health facilities. When asked on when one is expected to start FANC, 194(50.4%) of the respondents stated the fourth month of the pregnancy while 5.9% indicated second month, 13.2% in the first month while 13.5% reflected the third month of pregnancy. Regarding start of ANC at first trimester, the level of awareness was higher in urban facilities (36.6%) than rural facilities (9.5%). About 62% of the respondents knew that four FANC were recommended.

### Proportion of pregnant women utilizing FANC services

A total of 55.4% of the respondents were on antenatal visit while 21.5% were postnatal mothers, the rest 23.1% either came for family planning or when the child was sick. In the multivariable analysis, poor pregnancy outcome, low birth weight and still births were associated with failure to attend four or more ANC visits (OR=14.76, 95% CI 0.98, 223.17, p=0.05). For those attending post-natal clinic, 88.9% had live births. About 68% of respondents started the clinic visits at the second trimester of their pregnancy, 15.7% at first trimester and 14.7% at third trimester. Four and more ANC visits was associated with early ANC visits (p<0.001). About 12% of the women made more than 4 minimum visits for FANC services, 32% of the respondents made at least 4 visits, 20% attended 3 times, 17% attended 2 times while 12% attended once before delivery. About 6% could not be classified. The highest number of visits during pregnancy was recorded at rural (72.2%) health facilities compared to urban (27.8%) health facilities.

### Factors that influence timing in the uptake of FANC services

While looking at distance to the health facility, most of the respondents (49.1%) took 1-2hr walk to each health facility followed by 30mins walk at 30.1% (Table 1) as one of the hindrances to reaching the health facilties. Approximately 60.3% of the respondents reported to pay an amount between Kshs 100-200 (USD 1-2) while 22.9% paid less than Kshs 100 (USD 1) for laboratory tests during first ANC visits, which was found to be affordable given the number of tests. About 89.7% of respondents were satisfied with the services received at FANC. Dissatisfaction may have stemmed from walking up and down (32.4%) the health facilities for services and unavailability of services; 6.3% stated health care workers negative attitude (6.3%) and; 4.7% mentioned unavailability of drugs as reasons for dissatisfaction at FANC. More satisfaction was registered among HCWs manning rural (91.4%) health facilities than those in the urban health facilities (84.6%).

**Table 1:** Time taken by the women to reach a health facility

Distance	Frequency (n=385)	Percentage	Urban (MDH)	Rural Facilities
30 minutes walk	118	30.65%	28(29.8%)	84(30.9%)
1-2 hour walk	189	49.09%	33(35.1%)	147(54.0%)
30 minutes drive	46	11.95%	19(20.2%)	24(8.8%)
1-2 hour drive	13	3.38%	8(8.5%)	4(1.5%)
1-2 hour ride	6	1.56%	1(1.1%)	5(1.8%)
More than 2hr ride	13	3.38%	5(5.3%)	8(2.9%)
Total	385	100.0%	94(29.4%)	272(70.6%)

When asked about service delivery, most of the respondents said 1 hour (38.7%). Multivariable analysis to ascertain the independent predictors for dissatisfaction with FANC services showed the following 2 reasons emerged: Long waiting periods (2hrs) (OR=3.21,95% CI 1.03-10.01, p=0.045) and failure to get all the services (OR=11.51, 95% CI 4.68-28.29, p<0.001) as significant predictors. Clients attending rural health facilities took more time than those receiving services from urban facilities (Table 2).

**Table 2:** Time taken to receive focused antenatal care services

Waiting Time	Urban	Rural
30min	32(22.7%)	109(77.3%)
45 min	20(27.4%)	53(72.6%)
1 hr	10(15.15%)	56(84.85%)
2 hrs	11(23.9%)	35(76.1%)

Visiting the ANC clinic at least four time was not associated with the type of health facility (?2= 2.1545, p=0.142). Those with secondary education (OR=0.07, 95% CI 0.03- 0.18, p<0.001) or tertiary (OR=0.09, 95% CI 0.03-0.17, p<0.001) level education were less likely to go to rural health facility compared to those who have never schooled. Unemployed women were more likely to seek ANC services in a rural health facility compared to those on formal employment (OR=18.4 95% CI 5.3762.8, p<0.001) rather than MDH. Compared to Catholics, Muslims were less likely to attend a rural health facility (OR=0.25 95% CI 0.10-0.62, p=0.003).

## Discussion

The mean age of the respondents was 25 years. This gives an impression that most of the mothers were still in their youth which agrees with that, mother´s age plays an important role in utilization of maternal and child health services [9]. Majority (86.2%, Urban 75.3% Rural 90.8%) of the clients were found to be married, consistent with the literature on married women are more likely to utilize ANC services than single mothers. Distance affects up to four clinic attendance and above especially in the rural area compared to urban (as shown in table two below) [10]. There was an association between spouse’s level of education and level of awareness on FANC (p=0.03), for tertiary education. This was due to the fact that the level of education was related to level of income and most of them did not know the husbands monthly income. Majority of the respondents’ partners were low income earners meaning that they were less likely to use antenatal care services than those with higher economic status [11].

Existing literature on ANC has scarce information on cultural practices, attitude of clients and service providers, distance to the ANC clinic and policy instruments. During KIIs cultural/ traditional factors that were highlighted was that a real woman (“mche jeri”) does not have to seek medical attention during pregnancy because pregnancy is not a disease. Sometimes women acquire an antenatal care booklet in case need arises for them to deliver in the hospital because it will be asked by the hospital staff. This corroborates a similar finding done in Uganda [12]. Moreover religion, locally referred to “Imani moja” (one faith) was found to be a factor since as it required one not seek medical attention but to rely on God to heal them. This was consistent with other studies where religion was found to be a determinant of utilization of ANC services [13].

The impact of religion in determining MHCS utilization lies in the fact that it plays a significant role in shaping beliefs, norms and values including those that relate to childbirth and health services use. For example, it is argued that Islamic injunctions which encourage male domination constrain women´s power and autonomy which could limit the ability to make important decisions and also restrict movement [14].

Several factors were found to be associated with awareness in FANC. Christians demonstrated more awareness of FANC. Being a Muslim was protective. This was due to the fact that in the Islamic faith reproductive health subject was considered a subject not to be discussed easily between husband and wife [14], The study also found out that lack of awareness was arising from being unemployed (OR=9.94, 95% CI, p=0.005) which was related to low level of education or none.

Majority of the mothers (225, 62%) were of the opinion that four visits are recommended for focused antenatal care model while (180, 50.4%) began the visits in the fourth month of the pregnancy. This could be explained by Blooms theory on Taxonomy of objectives [15]. There was a high level of recommendation by the respondents for use of FANC. With this perceived high recommendation for services, utilization is yet to reach 100%.

The study found out that majority of the respondents, 68% start the ANC visits at the second trimester of their pregnancy. This was attributable to the low level of awareness and perceived wellness by the mothers. The study also found out that, 32% make four visits to the health facility for ANC. Results from the KIIs determined that most of them are aware of FANC (91%), the benefits, risks and even the number of visits they are supposed to make however some of them “feel lazy to attend” because of either distance, transport fees or they are not feeling sick. This study found out that poor pregnancy outcome which include low birth weight and still births was associated with failure to attend four or more ANC visits (OR=14.76, 95%CI p=0.05) which corroborates a previous study by Kaisa, Nona and Seppo, 2007 [16]. This study further established that there was an association between starting the ANC visits early (p<0.001) and completing the stipulated four visits. This was because early gestational age at the first visit gave the mother ample opportunity and increased awareness to attend up to five follow up visits [17]. For those utilizing the FANC services, there was no association between the type of health facility and utilization. This might have been due to the fact level of awareness was low in the rural areas.

Study results revealed that that 60.3% of the mothers paid an amount of between Kshs 100 – 200 (USD 1-2) for ANC laboratory tests. This was contrary to the findings of a study conducted in Uganda which indicated that demand side barriers such as transport and cost of maternal health services are a major challenge affecting utilization [18]. It was also found out that long waiting hours (1-2hrs), OR=3.21, 95% CI (1.03, 10.01), p=0.045), unavailability of services (mostly laboratory services) (OR=11.51, 95% CI (4.68, 28.29), p<0.001) and movements up and down (p<0.001) the health facility were the most common complaints for dissatisfaction. This was perceived as due to inadequate staff training, shortage of equipment/supplies and inappropriate infrastructure.

However, it was found out that those who took 30 minutes to be served (40.9%) were more likely to attend up to four visits. This was a motivation and considered as an enabling accessibility factor [19].

During the KIIs poor communication from respondents encountered by health workers came out as factor since they would wait for so long not knowing what was happening hence one said; “Kama mtoto wake ni mgonjwa sisi ni wamama tunaelewa tunaweza kumsaidia badala wanyamaze na sisi tunangoja tu wangetuambia twende nyumbani tuje kesho. (As women, we also also understand when their(health care workers) children are sick and instead of keeping us waiting, we would not have an objection if told us to go and come back tomorrow)”.

Because of the delays encountered mothers go to private facilities and they use new books. It was also found out that health education was not comprehensive enough due to inadequate staffing and the long queues.

## Conclusion

Utilization of FANC services shown through completion of four ANC visits in both rural and urban health facilities was low. The level of awareness on the recommended number of visits and FANC services was associated with religion, occupation of mothers, their education level and that of their spouses. There is need to upscale health promotion initiatives on maternal, newborn and child health. Distance from the facility, employment status, religion, age, spouse’s education and occupational status and negative culture/traditions were the main demographic factors associated with utilization of FANC. Health promotions sessions among women should be done to enhance individual birth plans. Household chores including distance from water source were not a hindrance to ANC attendance. Long waiting hours, disharmony in the hospital departments, lack of all services were the major causes of dissatisfaction with health facility services. There is therefore need to standardize services across the health facilities in both rural and urban areas.

### What is known about this topic

- Religion affects the utilization of services among those of Muslim faith;
- Demand side barriers such as transport and cost of maternal health services are a major challenge affecting utilization;
- Poor pregnancy outcome which include low birth weight and still births was associated with failure to attend four or more ANC visits.

### What this study adds

- Supply side factors that affect utilization of these factors are interdepartmental disharmony, lab services, ANC, PNC etc not being streamlined which may not be related to adequate staff and quality facilities and equipment as other studies have found out;
- Cultural/traditional factors that were highlighted was that a real woman (“mche jeri”) does not have to seek medical attention during pregnancy because pregnancy is not a disease, a barrier that maybe unique for this study area.
